# Organophosphorus and Carbamate Pesticide Residues Detected in Water Samples Collected from Paddy and Vegetable Fields of the Savar and Dhamrai Upazilas in Bangladesh

**DOI:** 10.3390/ijerph9093318

**Published:** 2012-09-11

**Authors:** Md. Alamgir Zaman Chowdhury, Sanjoy Banik, Borhan Uddin, Mohammed Moniruzzaman, Nurul Karim, Siew Hua Gan

**Affiliations:** 1 Agrochemicals and Environmental Research Division, Institute of Food & Radiation Biology, Atomic Energy Research Establishment, Savar, Dhaka 1349, Bangladesh; Email: sanjoy.bmb319@gmail.com (S.B.); rasmo04@yahoo.com (M.M.); 2 Department of Biochemistry and Molecular Biology, Jahangirnagar University, Savar, Dhaka 1342, Bangladesh; Email: borhan.masud@gmail.com (B.U.); nkripon@yahoo.com (N.K.); 3 Department of Pharmacology, School of Medical Sciences, Universiti Sains Malaysia, Kubang Kerian, Kelantan 16150, Malaysia; 4 Human Genome Centre, School of Medical Sciences, Universiti Sains Malaysia, Kubang Kerian, Kelantan 16150, Malaysia; Email: shgan@kck.usm.my

**Keywords:** pesticides, organophosphorus, carbamate, HPLC

## Abstract

Several types of organophosphorous and carbamate pesticides have been used extensively by the farmers in Bangladesh during the last few decades. Twenty seven water samples collected from both paddy and vegetable fields in the Savar and Dhamrai Upazilas in Bangladesh were analyzed to determine the occurrence and distribution of organo-phosphorus (chlorpyrifos, malathion and diazinon) and carbamate (carbaryl and carbofuran) pesticide residues. A high performance liquid chromatograph instrument equipped with a photodiode array detector was used to determine the concentrations of these pesticide residues. Diazinon and carbofuran were detected in water samples collected from Savar Upazila at 0.9 μg/L and 198.7 μg/L, respectively. Malathion was also detected in a single water sample at 105.2 μg/L from Dhamrai Upazila. Carbaryl was the most common pesticide detected in Dhamrai Upazila at 14.1 and 18.1 μg/L, while another water sample from Dhamrai Upazila was contaminated with carbofuran at 105.2 μg/L. Chlorpyrifos was not detected in any sample. Overall, the pesticide residues detected were well above the maximum acceptable levels of total and individual pesticide contamination, at 0.5 and 0.1 μg/L, respectively, in water samples recommended by the European Economic Community (Directive 98/83/EC). The presence of these pesticide residues may be attributed by their intense use by the farmers living in these areas. Proper handling of these pesticides should be ensured to avoid direct or indirect exposure to these pesticides.

## 1. Introduction

Agriculture is the largest economic sector in Bangladesh, representing 23.50% of the gross domestic product, and contributing to nearly half of the country’s economic output [1]. Approximately 70% of the population is involved in agriculture, with a total crop production of approximately 27.79 million metric tons [2]. However, flooding, drought, thunderstorms, attacks from pests and diseases on crops and vegetables can dramatically reduce agricultural output. It has been reported that 20% of agricultural products in Bangladesh are destroyed every year both in the field and in storage by these occurrences [3]. Therefore, like many other developing countries, pesticides are used extensively in Bangladesh to increase the crop yield per acre [4,5]. Due to the widespread use of pesticides, their residues are detected in various environmental matrices, including soil, water and air. Moreover, the presence of pesticides in the environment has caused great social and scientiﬁc concern all over the World [6].

Over the years, various types of pesticides, such as organochlorine, organophosphorous and carbamate ones have been extensively used by farmers in Bangladesh [4]. Since organochlorine pesticides have been banned in 1993 according to the Bangladesh Environment Conservation Act 1995 due to their high toxicity [7,8], organophosphorous pesticides are widely used in agriculture. In Bangladesh, it is estimated that up to 64% of the crop-producing area is treated with carbamates, while up to 35% of the crop-producing area is treated with organophosphates [9].

The effectiveness of organophosphorous pesticides, coupled with their relatively cheap cost encourages farmers to use more of these pesticides when growing their field crops. Yet, these pesticides pose severe risks to the farmers’ health. The pesticides’ residues are discharged into the air and water. Through the consumption of foods containing these pesticides at a level of 0.1 µg/L, these residues can affect the human body [10]. The widespread use of pesticides may contaminate the environment and freshwater fish [11], which ultimately are consumed by humans. Moreover, moderate to severe respiratory and neurological damage can be caused by many of these compounds, which are genotoxic and carcinogenic [12]. In trace amounts, chlorpyrifos has been reported to cause neurological disorders such as attention deficit hyperactivity disorder and a developmental disorder both in fetuses and children [13]. Furthermore, carbofuran, which is a carbamate, has been reported to cause serious reproductive problems, while occupational exposure to carbaryl has been reported to result in nausea, vomiting, blurred vision, coma and difficulty in breathing [14,15].

In short, there is an indiscriminate use of pesticide in Bangladesh, so pollution of environmental resources through the use of pesticides is very likely. Although there are Pesticide Acts and Rules (The Environment Court Act, 2000) [8], some important provisions of the legislation are not strictly adhered to, which may result in the gradual increase in the risk to humans, animals, fish, birds and the environment. Currently, there are no strict restrictions of pesticide use on vegetables and crops. As a result of ignorance, some farmers also do not observe the waiting period after spraying pesticides due to the lack of knowledge. Even worse, when the desired effects are not achieved, farmers tend to increase the dose to higher levels. Such indiscriminate use of pesticides can result in the accumulation of toxic residue in food products, which may ultimately cause human health complications [16]. Therefore, using pesticides while maintaining good water quality is an immense challenge [17] due to the persistence of pesticides in water.

The situation is very similar in Savar and Dhamrai Upazilas in Dhaka, Bangladesh, where various types of pesticides are used extensively by the farmers in considerable quantities. This is because agriculture is the main activity in both Dhamrai Upazila (41.77%) [18] and in Savar Upazila (24.34%) [19]. These two districts or “upazilas” are among the few main sources of vegetables and crops for the capital city of Dhaka, Bangladesh, which produce mainly paddy crops. The safe and effective use of these pesticides is essential, to avoid contamination of the water and soil environments of these regions. The objective of this study was to determine the presence organophosphorus and carbamate pesticide residues in these two regions so that the inhabitants as well as the farmers of this region will be informed about the level of pesticide exposure and the quality of water in their environment.

## 2. Experimental Section

### 2.1. Chemicals and Reagents

Chlorpyrifos (99.0%), carbofuran (99.5%), carbaryl (98.5%), malathion (97.5%) and diazinon (99.0%) standards (Table 1) were of reference grade and were purchased from Dr. Ehrenstorfer GmbH, D-86199 Augsburg, Germany. The solvents, such as acetone (BDH, England), *n*-hexane (Merck, Germany), and diethyl ether (BDH, UK) were of analytical grade, while acetonitrile (Scharlau, Barcelona, Spain) was of HPLC grade.

**Table 1:** Structures, chemical properties, functions, DT_50 _and LD_50 _of the pesticides investigated in this study.

Compound	Formula and CAS No	Structure	Chemical Class and Function	DT_50_ (days)	LD_50_ (mg/ kg)
**Chlorpyrifos**	C_9_H_11_ Cl_9_NO_3_PS; 2921-88-2	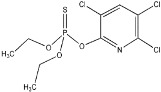	Organophosphorus; Insecticide, nematicide	16–72	Rats: 95–270
**Malathion**	C_10_H_19_O_6_PS_2;_ 121-75-5	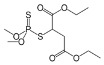	Organophosphorus; Insecticide	0.49–107	Rats: 1,522–1,945
**Diazinon**	C_12_H_21_N_2_O_3_PS; 333-41-5	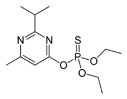	Organophosphorus; Insecticide	7–15	Rabbit: 1,160–1,340
**Carbofuran**	C_12_H_15_NO_3;_ 1563-66-2	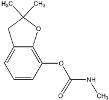	N-methyl carbamate; Insecticide, nematicide	13–14	Rats: 90–500
**Carbaryl**	C_12_H_11_NO_2;_ 63-25-2	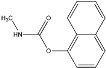	N-methyl carbamate; Insecticide, plant growth regulator, nematicide	0.15–35	Rats: 50–500

**DT_50_**: degradation time for 50% of a compound; **LD_50_**: lethal dose for 50% of the population.

### 2.2. Collection and Preservation of Water Samples

**Figure 1:**
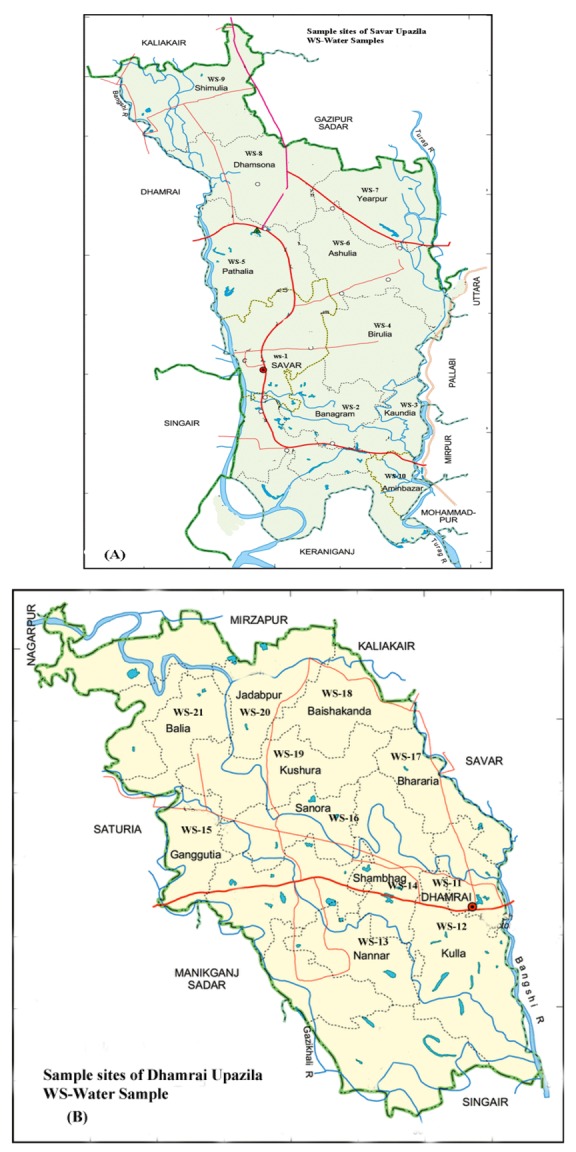
Sample sites of Savar (**A**) and Dhamrai Upazila (**B**).

Surface water samples (n = 27) from both the paddy and vegetable fields of Savar (n = 16) and Dhamrai Upazilas (n = 11), in Dhaka, Bangladesh were collected from May–July, 2009 (Figure 1). The samples were kept in clean amber glass bottles, put into ice boxes and immediately transferred to the laboratory at the Institute of Food and Radiation Biology, Bangladesh Atomic Energy Commission, Savar, Dhaka. Sample collection was performed according to the recommendations by Hunt and Wilson [20] and APHA [21]. The samples were stored at −20 °C prior to analysis.

A brief survey was also conducted at the location of the sample collection. Questionnaires were randomly administered to the farmers for gathering information on the types of pesticide commonly used, to verify the findings and also to determine if there was any unauthorized or authorized pesticide use by the farmers in the two districts.

### 2.3. Sample Extraction

Five hundred mL water samples were transferred to a 1,000 mL capacity separating funnel before extraction, using 100 mL of solvent mixture of 2% diethyl ether in double-distilled *n*-hexane. The organic solvent was collected in a conical flask. This was followed by two further extractions with 25 mL of solvent mixture using a similar procedure. The organic solvent layers were aspirated and combined before the addition of 20 g of anhydrous sodium sulfate (Merck, Germany), to remove the residual water. The solvent was then rotary vacuum evaporated (Buchi, Switzerland) to a smaller volume of 5 mL, based on the method described in the Deutsche Forschungsgemeinschaft [22].

The concentrated samples were passed through a column (10 mm ID) packed with 10 g of deactivated florisil (Sigma, USA) and synthetic magnesium silicate (60–100 mesh). The top 1.5 cm of the florisil column was packed with anhydrous sodium sulfate. This extract was eluted with 2% diethyl ether (double distilled using a fractional distillation plant, Schott Duran, Germany) in *n*-hexane (double distilled) at 5 mL/min. The eluent was further concentrated in a rotary vacuum evaporator (Buchi, Switzerland) before being transferred to a glass vial. The solvent was completely dried under a gentle nitrogen flow. The dried sample was reconstituted in 1 mL of ACN for a subsequent analysis using high performance liquid chromatography (HPLC).

### 2.4. Positive Controls

Four hundred micro-liters of chlorpyrifos, carbofuran, carbaryl, malathion and diazinon standards (100 ng/µL) were added to 500 mL of blank water samples in triplicate as a positive control. The standards were left standing for 10 min to produce a better interaction with the samples. Subsequently, the fortified sample volume was processed to 2 mL, as mentioned in the “extraction and clean-up section of materials and methods”, before their injection into the HPLC.

### 2.5. HPLC Analysis

Following the sample cleanup, aliquots of the final volume were quantified using an HPLC (Shimadzu, Japan) LC-10 ADvp, equipped with an SPD-M 10 Avp attached to a photo-diode array detector (PDA) (Shimadzu SPD-M 10 Avp, 200–800 nm). The analytical column was a C18 Reverse Phase Alltech (250 × 4.6 mm, 5 µm) that was maintained at 30 °C in a column oven. The mobile phase, a combination of 70% ACN and 30% water, was filtered using a cellulose filter of 0.45 µm before each use. The flow rate was 1.0 mL/min, and all solvents used were of HPLC grade.

**Figure 2:**
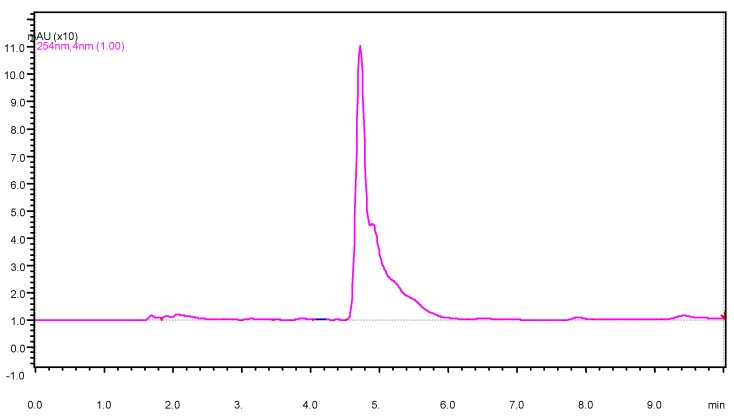
Typical chromatogram of a carbaryl standard injected at 60 µg/mL (Retention time 4.73 min).

Prior to HPLC analysis, the samples were passed through 0.45 µm of nylon (Alltech Associates, IL, USA) syringe filters. The 20 µL samples were manually injected each time. The identification of the suspected pesticide was performed, relative to the retention time of the pure analytical standard. Quantification was performed based on the method described by [9]. A typical chromatogram from the analysis is shown in Figure 2.

#### 2.5.1. Calibration Curve

The calibration curves for chlorpyrifos, carbofuran, carbaryl, malathion and diazinon were prepared at four concentrations of 0, 5, 10, 20 and 40 µg/L (R^2^ = 96.46).

#### 2.5.2. Recovery

The mean percentage recoveries for the various pesticides were calculated using the following equation:


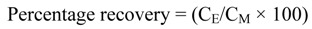


where C_E_ is the experimental concentration determined from the calibration curve and C_M_ is the spiked concentration.

## 3. Results

Twenty seven surface water samples were randomly collected from paddy and vegetable fields. The levels were analyzed for four organophosphorus and two carbamate pesticide residues and compared with the guidelines and limits set by the European Economic Commission (EEC) (Directive 98/83/EC). The mean percentage recoveries of chlorpyrifos, carbofuran, carbaryl, malathion and diazinon in the spiked positive controls of the water samples with the florisil cleanup system were 87.75%, 85.00%, 93.75%, 81.25% and 96.38%, respectively (Table 2).

**Table 2:** Percentage recoveries of chlorpyrifos, carbofuran, carbaryl, malathion and diazinon.

Compound	Clean up system	Amount (ng) in HPLC *		Recovery %
		Spiked	Measured	
**Chlorpyrifos**	Control	0	0	0
	Florisil clean up	400.00	351.00	87.75
**Carbofuran**	Control	0	0	0
	Florisil clean up	400.00	340.00	85.00
**Carbaryl**	Control	0	0	0
	Florisil clean up	400.00	375.00	93.75
**Malathion**	Control	0	0	0
	Florisil clean up	400.00	325.00	81.25
**Diazinon**	Control	0	0	0
	Florisil clean up	400.00	385.50	96.38

* Mean value of triplicates.

Diazinon and carbofuran were detected in water samples collected from Savar Upazila at 0.9 and 198.7 μg/L respectively (Table 3). Malathion was also detected in a single water sample from Dhamrai Upazila at 105.2 μg/L. Carbaryl was the most common pesticide detected in Dhamrai Upazila at 14.1 (Figure 1) and 18.1 μg/L, while carbofuran was detected in another water sample from Dhamrai Upazila at 105.2 μg/L. Chlorpyrifos was not detected in any sample.

**Table 3:** Concentrations of Organophosphorus and Carbamate Pesticide Residues in Water Samples of Savar and Dhamrai Upazila.

Sample ID	Organophosphorus Pesticide Residues (µg/L)		Carbamate Pesticide Residues (µg/L)	
	Malathion	Diazinon	Carbaryl	Carbofuran
WS-13	ND	ND	**14.1**	ND
WS-17	ND	ND	**18.1**	ND
WS-20	**105.2**	ND	ND	**105.2**
WS-23	ND	**0.9**	ND	ND
WS-25	ND	ND	ND	**198.7**

Table showing only WS that contained pesticides residues; organophosphorus and carbamate pesticide residues were not detected in the rest of the samples. WS: Water Sample; ND: Not Detected; WS-17 and WS-20 are samples from Dhamrai Upazila; WS-23 and WS-25 are samples from Savar Upazila. Mean value of triplicates. LOD: 0.01 µg/L. Concentrations in bold are those that exceed levels that are safe for humans, established by the EEC at 0.1 µg/L for any pesticide (or 0.5 µg/L for total pesticides).

## 4. Discussion

This is the first study that reveals the occurrence and distribution of organophosphorus and carbamate pesticide residues in samples originating from the paddy and vegetable fields of Savar (n = 16) and Dhamrai Upazila (n = 11) in Bangladesh. The results of this study showed that organophosphorus and carbamate pesticide residues were detected at a higher concentration in some of the water samples. The highest concentration of malathion pesticide detected in our study, at 105.2 µg/L, was found in water samples from a paddy field of Dhamrai, which exceeded by 1,052 times the allowable limit of 0.1 µg/L of pesticide contamination set by the EEC (Directive 98/83/EC). Moreover, high levels of carbofuran pesticide residues of 105.2 and 198.7 µg/L were detected in two of the water samples collected from Savar Upazila.

There are two possible reasons for high levels of pesticides. First, the pesticides are used to kill both insects and nematodes, to ensure the proper growth of paddy and vegetables. Secondly, most of the farmers in the study area may not have enough knowledge about the chemical nature of pesticides that have been used or the effects of pesticides on the environment and the exposure effects of pesticides on public health, when using them indiscriminately. Therefore, it is advised that people living in this area do not consume fish or other aquatic animals caught in paddy fields because aquatic animals may be contaminated by these pesticides. It is also possible that detrimental residues may remain in the edible portion of vegetables or plants and may affect humans because humans are at the top of the food chain and are, therefore, most susceptible [23].

Chlorpyrifos was not detected in any of the samples during the present investigation. Diazinon was only detected in a single water sample collected from the Ashulia Union of Savar Upazila at 105.2 μg/L. This level is approximately 50 times higher than the levels detected in a water sample collected from the Ganges River in India at 2.61 μg/L [24], which can be attributed to the extensive and uncontrolled agriculture use of pesticide in Dhamrai Upazila.

Malathion was also present in a single water sample from the Bhararia Union of Dhamrai Upazila at 105.2 μg/L. Diazinon was only detected in a single water sample collected from the Ashulia Union of Savar Upazila in the Dhaka District. The level of diazinon was 0.9 μg/L, which is greater than the normal level of pesticide of 0.1 µg/L, set by the EEC (Directive 98/83/EC). This level is lower than the highest level (1,140 μg/L) of diazinon detected in water samples one day after spraying was conducted in Iran [25].

**Figure 3:**
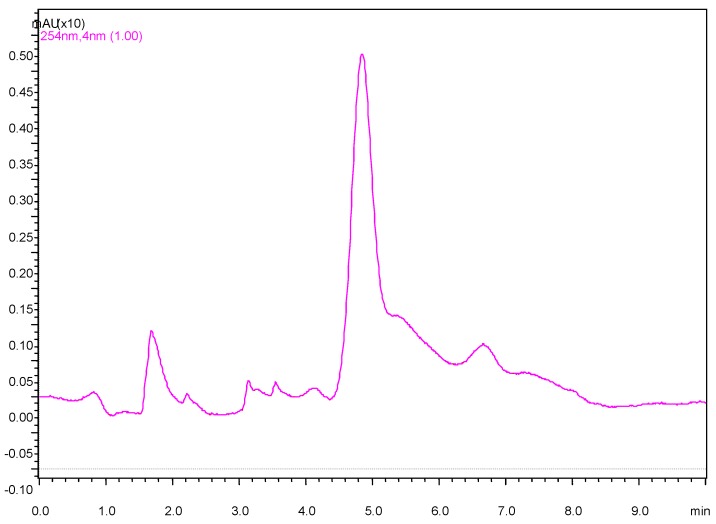
Chromatogram of WS-13 showing the presence of carbaryl (Retention time 4.84 min).

From our study, carbaryl and carbofuran were the two most commonly identified pesticides that were also detected at much higher levels than the allowed level of pesticide, set by the EEC (Directive 98/83/EC) at 0.1 µg/L. For example, carbaryl was detected at 14.1 μg/L (Figure 3) and 18.1 μg/L in the two water samples of Dhamrai Upazila,, although this pesticide was detected at a much lower level of 0.163 μg/L in a water sample of a paddy field in Rangpur, Bangladesh [9] or at 3.78 μg/L in a water sample collected from USA [26]. This indicates that two pesticides are commonly utilized in these areas. The result also correlates with our survey data, which confirmed that most of the farmers claimed to have used *Furadan*^®^ (carbaryl) in the investigated areas. In addition to carbaryl, the farmers also admitted using chlorpyrifos, malathion, diazinon and carbofuran, although chlorpyrifos was not detected in any of the samples. However, it is possible that this fact was attributed by its short persistence time.

Carbofuran was detected in the samples collected from the Ashulia Union of Savar Upazila at 105.2 and 198.7 μg/L respectively. In a previous study, carbofuran was detected at a lower concentration of 3.395 μg/L in a sample collected from the paddy fields in Rangpur, Bangladesh [9]. It is possible that carbofuran is used in large quantities in Savar Upazila. Carbofuran level was reported to be higher (0.01 mg/L–0.592 mg/L) in river and pond water samples collected from Kenya [27].

Organochlorine pesticides (OCPs) are persistent contaminants in the environment that are of great concern because of their persistent and long-range transportable nature as well as toxic biological effects [28,29,30]. Due to a long persistence time in nature, organochlorine pesticides have not been used in agricultural practices in recent years. Therefore, they are less likely to be detected. As a result, we have not analyzed the samples for the presence of organochlorine pesticide residues. However, the use of organophosphorus and carbamate pesticides such as chlorpyrifos, diazinon, malathion, carbofuran and carbaryl has greatly increased because of their less detrimental effects on the environment, resulting from a small persistence time (2 h to 8 weeks).

It is recommended that more water samples should be collected at different time intervals in future studies because the nationwide pattern of pesticide use varies annually. A declining trend in pesticides levels has been reported in water samples analysed on the first day of spraying when compared with those collected two months later [25] indicating the importance of proper recording of the application time and doses of pesticides. Because a number of samples from Savar and Dhamrai Upazilas were highly contaminated with pesticide exceeding safe levels, water samples from this area need to be monitored on a routine basis to ensure safety.

## 5. Conclusions

The present study reports that 22% of water samples collected from the paddy and vegetable fields of Savar and Dhamrai Upazilas are highly polluted with pesticide residues, particularly with carbaryl. Other pesticides detected were malathion, diazinon and carbofuran. In general, the levels were higher than those reported from other countries. The presence of these pesticide residues may be attributed by their intense use by the farmers living in these areas. Proper handling of these pesticides should be ensured to avoid direct or indirect exposure to these pesticides. New and strict enforcement should be implemented by the authorities as soon as possible in this region of Bangladesh to control the indiscriminate use of pesticides.

## References

[1] Islam M., Shamsad S. (2009). Assessment of irrigation water quality of Bogra district in Bangladesh. Bangladesh J. Agric. Res..

[2] West G. Food and Agriculture in Bangladesh: A Success Story. http://scholar.googleusercontent.com/scholar?q=cache:k6o55zmknmMJ:scholar.google.com/+Food+and+agriculture+in+Bangladesh:+A+success+story&hl=zh-CN&as_sdt=0&as_vis=1.

[3] Sallam M.N. Insect Damage: Damage on Post-Harvest. http://www.fao.org/fileadmin/user_upload/inpho/docs/Post_Harvest_Compendium_-_Pests-Insects.pdf.

[4] Dasgupta S., Meisner C., Huq M. (2007). A pinch or a pint? Evidence of pesticide overuse in Bangladesh. J. Agric. Econ..

[5] (1993). Practical Approaches to Crop Pest and Disease Management in Bangladesh.

[6] Rodrigues A.M., Ferreira V., Cardoso V.V., Ferreira E., Benoliel M.J. (2007). Determination of several pesticides in water by solid-phase extraction, liquid chromatography and electrospray tandem mass spectrometry. J. Chromatogr. A.

[7] Matin M., Malek M., Amin M., Rahman S., Khatoon J., Rahman M., Aminuddin M., Mian A. (1998). Organochlorine insecticide residues in surface and underground water from different regions of Bangladesh. Agric. Ecosyst. Environ..

[8] National Integrated Pest Management Policy, B. Distribution and Use of Pesticides in Bangladesh. http://www.doe-bd.org/pop/pdf/Report%20Text%20Final.pdf.

[9] Chowdhury A.Z., Jahan S.A., Islam M.N., Moniruzzaman M., Alam M.K., Zaman M.A., Karim N., Gan S.H. (2012). Occurrence of organophosphorus and carbamate pesticide residues in surface water samples from the Rangpur district of Bangladesh. Bull. Environ. Contam. Toxicol..

[10] Wang D.F., Sun J.P., Du D.H., Sun L.P., Chen Z.D., Xue C.H. (2007). Degradation of extraction from seaweed and its complex with rare earths for organophosphorous pesticides. J. Rare Earths.

[11] Rahman M.M. Pestcides: Their Uses and Problems in Context of Bangladesh. Proceedings of the National Workshop on Conventional and Nuclear Technique for Pesticide Residues Studies in Food and Environment at IFRB.

[12] Hayat K., Ashfaq M., Ashfaq U., Saleem M.A. (2011). Determination of pesticide residues in blood samples of villagers involved in pesticide application at district Vehari (Punjab), Pakistan. Afr. J. Environ. Sci. Technol..

[13] Rauh V.A., Garfinkel R., Perera F.P., Andrews H.F., Hoepner L., Barr D.B., Whitehead R., Tang D., Whyatt R.W. (2006). Impact of prenatal chlorpyrifos exposure on neurodevelopment in the first 3 years of life among inner-city children. Pediatrics.

[14] U.S. Environmental Protection Agency (EPA) (1984). Health and Environmental Effects Profile for Carbaryl; EPA/600/x–84/155.

[15] U.S. Environmental Protection Agency (EPA) (1989). Pesticide Fact Sheet Number 199: Cypermethrin.

[16] Nasreddine L., Parent-Massin D. (2002). Food contamination by metals and pesticides in the European Union. Should we worry?. Toxicol. Lett..

[17] Gan J. Pesticide and Groundwater Quality. http://www.pw.ucr.edu/textfiles/PesticideWiseWinter2002.htm.

[18] Banglapedia Information about Dhamrai Upazila. http://www.banglapedia.org/httpdocs/HT/D_0182.HTM.

[19] Banglapedia Information about Savar Upazila. http://www.banglapedia.org/httpdocs/HT/S_0148.HTM.

[20] Hunt D.T.E., Wilson A.L. (1986). Reference. The Chemical Analysis of Water-General Principles and Techniques.

[21] (1995). Standard Methods for the Examination of Water and Waste Water.

[22] Thier H.P., Zeumer H. (1987). Manual of Pesticide Residue Analysis.

[23] Bakore N., John P., Bhatnagar P. (2004). Organochlorine pesticide residues in wheat and drinking water samples from Jaipur, Rajasthan, India. Environ. Monit. Assess..

[24] Sankararamakrishnan N., Kumar Sharma A., Sanghi R. (2005). Organochlorine and organophosphorous pesticide residues in ground water and surface waters of Kanpur, Uttar Pradesh, India. Environ. Int..

[25] Arjmandi R., Tavakol M., Shayeghi M. (2010). Determination of organophosphorus insecticide residues in the rice paddies. Int. J. Environ. Sci. Technol..

[26] Bushway R. (1981). High-performance liquid chromatographic determination of carbaryl and 1-naphthol at residue levels in various water sources by direct injection and trace enrichment. J. Chromatogr. A.

[27] Otieno P.O., Lalah J.O., Virani M., Jondiko I.O., Schramm K.W. (2011). Carbofuran use and abuse in Kenya: Residues in soils, plants, water courses and the African white-backed vultures (*Gyps africanus*) found dead. Environmentalist.

[28] Barri T., Bergstrom S., Hussen A., Norberg J., Jonsson J.Ã. (2006). Extracting syringe for determination of organochlorine pesticides in leachate water and soil-water slurry: A novel technology for environmental analysis. J. Chromatogr. A.

[29] Gao J., Liu L., Liu X., Lu J., Zhou H., Huang S., Wang Z., Spear P.A. (2008). Occurrence and distribution of organochlorine pesticides-lindane, p, p’-DDT, and heptachlor epoxide-in surface water of China. Environ. Int..

[30] Geyikçi F., Büyükgüngör H. (2011). Monitoring of organochlorine pesticides in the surface waters from mid-Black sea region, Turkey. Environ. Monit. Assess..

